# The Effects of a Whole-Food Plant-Based Nutrition Education Program on Blood Pressure and Potassium in Chronic Kidney Disease: A Proof-of-Concept Study

**DOI:** 10.3390/nu17050779

**Published:** 2025-02-24

**Authors:** Scott E. Liebman, Andrea Baran, Ted D. Barnett, Thomas M. Campbell, Luojing Chen, Susan M. Friedman, Shamsul Hasan, Thu H. Le, Rebeca D. Monk, Janany Sabescumar, Nellie Wixom, Anne Zhang, Erin K. Campbell

**Affiliations:** 1 Department of Medicine, University of Rochester Medical Center, Rochester, NY 14642, USA; luojing_chen@urmc.rochester.edu (L.C.); susan_friedman@urmc.rochester.edu (S.M.F.); thu_le@urmc.rochester.edu (T.H.L.); rebeca_monk@urmc.rochester.edu (R.D.M.); janany_sabescumar@urmc.rochester.edu (J.S.); anne_zhang@urmc.rochester.edu (A.Z.); 2 Department of Biostatistics and Computational Biology, University of Rochester Medical Center, Rochester, NY 14642, USA; andrea_baran@urmc.rochester.edu; 3 Rochester Lifestyle Medicine Institute, Rochester, NY 14692, USA; ted.barnett@roclifemed.org; 4 Department of Family Medicine, University of Rochester Medical Center, Rochester, NY 14642, USA; thomas_campbell@urmc.rochester.edu (T.M.C.); erin_campbell@urmc.rochester.edu (E.K.C.); 5 Department of Medicine, University of Pittsburgh Medical Center, Pittsburgh, PA 15219, USA; hasans4@upmc.edu; 6 Clinical Research Center, University of Rochester Medical Center, Rochester, NY 14642, USA; nellie_wixom@urmc.rochester.edu

**Keywords:** chronic kidney disease, hypertension, hyperkalemia, plant-based diet, education

## Abstract

Background/Objectives: Whole-food plant-based diets (WFPBDs) are beneficial in managing hypertension in the general population but have not been well studied in chronic kidney disease (CKD), potentially due to concerns about hyperkalemia. We hypothesized that individuals with CKD 3 or 4 attending a 15-day WFPBD education program would achieve lower blood pressure compared to those who did not, without an increased risk of hyperkalemia. Methods: This was a pilot trial of 40 subjects with mild-to-moderate CKD and hypertension but without diabetes or proteinuria from a single academic center. The subjects were randomized to the 15-day education program or the control group. The changes in blood pressure, serum potassium, and other anthropometric and biochemical values were assessed. Results: Systolic blood pressure decreased from the baseline to day 15 in the intervention group by 8 mm Hg and increased in the control group by 2.7 mm Hg, although the difference in the blood pressure change did not reach statistical significance (*p* = 0.12). Diastolic blood pressure was not different between the two groups. Potassium changed by 0.01 mEq/L in the intervention group and −0.07 mEq/L in the control group (*p* = 0.52). The intervention subjects had significant decreases in body mass (−3.0 vs. −0.12 kg, *p* < 0.0001), total cholesterol (−39.4 vs. −5.0 mg/dL, *p* < 0.0001), low-density lipoprotein (−28.4 vs. −0.6 mg/dL, *p* < 0.0001), and high-density lipoprotein (−8.6 vs. −0.4 mg/dL, *p* = 0.006) compared to the controls. The changes in albumin and phosphorus were not different between the two groups. Conclusions: The subjects with mild-to-moderate CKD attending a 15-day WFPBD education program had a non-statistically significant reduction in systolic blood pressure without an increased risk of hyperkalemia compared to those who did not attend. The intervention subjects achieved significantly greater reductions in body mass and cholesterol without adverse effects on albumin or phosphorus. Larger and longer-duration trials using this approach in a diverse group of CKD patients are warranted.

## 1. Introduction

Chronic kidney disease (CKD) affects approximately 14% of adults in the United States [1] and is associated with increased rates of morbidity and mortality [1]. The mainstay of therapy to delay CKD progression is the management of risk factors, such as hypertension, diabetes, and proteinuria.

The United States Renal Data System data show that >90% of Medicare beneficiaries with CKD have concomitant hypertension [1]. Better blood pressure (BP) control in those with CKD is beneficial in delaying progression [2] and decreasing cardiovascular events [3,4] and mortality [3,5,6]. The Kidney Disease Improving Global Outcomes (KDIGO) Clinical Practice Guidelines for the Management of Blood Pressure in Chronic Kidney Disease suggest a systolic blood pressure (SBP) target of <120 mm Hg [7].

Diet and lifestyle changes are fundamental to hypertension management [8]. The Dietary Approach to Stop Hypertension (DASH) trial demonstrated that a diet rich in fruits, vegetables, and low-fat dairy foods significantly reduced BP in the general population [9]. There is growing interest in managing hypertension using whole-food plant-based diets (WFPBDs), which emphasize unprocessed plant-derived foods while minimizing or avoiding animal and processed plant products [10]. This approach has not been codified into the guidelines for blood pressure management in CKD patients. The KDIGO Clinical Practice Guidelines for the Management of Blood Pressure in Chronic Kidney Disease recommend sodium restriction but do not recommend the adoption of any specific diet [7], and dietary studies in those with CKD have yielded mixed results. A study examining the DASH diet in CKD did not show a benefit in lowering mean BP [11]. Conversely, Goraya et al. found that fruit and vegetable supplementation had a greater anti-hypertensive effect than bicarbonate supplementation or a placebo in individuals with CKD 4 and metabolic acidosis [12].

One concern of using WFPBDs in those with CKD is hyperkalemia. Further, in the dietary trials above [11,12], food was supplied to participants, which may not be practical on a larger scale.

Rochester Lifestyle Medicine Institute’s (RLMI) 15-day Jumpstart program (“Jumpstart”), built on the principles of Self-Determination Theory, uses a combination of lectures, support systems, and food demonstrations to help individuals adopt a WFPBD. A previous Jumpstart cohort showed a 6.8 mm Hg decrease in SBP and a 16.8 point drop in those with baseline hypertension [13]. As the Jumpstart program has not been tested in CKD patients, this pilot study tested the hypothesis that individuals with CKD stage 3/4 attending Jumpstart would achieve lower blood pressure without an increased risk of hyperkalemia compared to those who did not.

## 2. Materials and Methods

### 2.1. Study Design and Participants 

**Figure 1:**
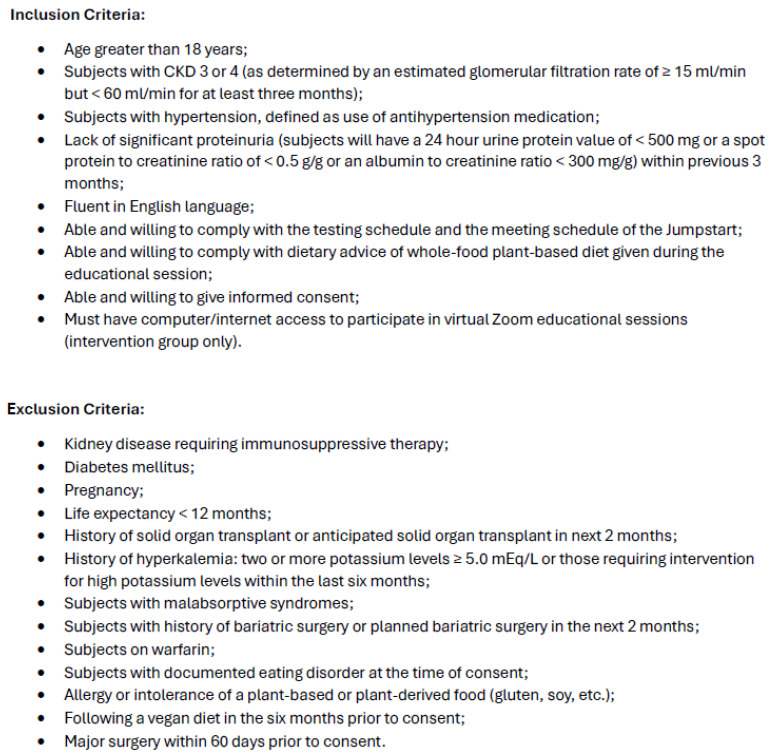
Inclusion and exclusion criteria.

The subjects were recruited from the University of Rochester’s nephrology clinics between November 2019 and December 2022. Figure 1 shows the inclusion and exclusion criteria. This study was approved by the Institutional Research Subjects Review Board (study 00003817), and the subjects provided written informed consent. The trial was registered at clinical trials.gov (NCT06343129, 1 April 2024). The research was conducted in accordance with the Declaration of Helsinki. The enrolled subjects were randomized to the Jumpstart program or control via Microsoft Excel (Microsoft, Redmond, WA, USA). The investigators were blinded to the participants’ group assignments. Due to the food-based and program-based nature of the intervention, blinding of the participants was not feasible.

### 2.2. Educational Intervention

The intervention group subjects participated in the 15-day Jumpstart program (https://rochesterlifestylemedicine.org/about-jumpstart/, accessed on 18 February 2025), administered by RLMI, a local not-for-profit organization.

The Jumpstart program was developed based on Caldwell Esselstyn’s Cardiovascular Disease Prevention and Reversal program at the Cleveland Clinic, which employs a low-fat WFPBD as the cornerstone of managing and reversing coronary heart disease [14].

Jumpstart is a 15-day online program that provides education and practical advice on why and how to consume a low-fat, plant-based diet for optimal health. The program does not prescribe a diet per se and does not provide food, but it does provide sample recipes and optional shopping lists. The individuals taking the program are instructed to eat from 4 main food groups over the two-week period: vegetables, fruits, whole grains, and legumes. The participants are advised to exclude all animal products, high-fat plant foods (such as oils, nuts, nut butters, seeds, avocados, olives, and coconut), and processed foods, including those with added oil or sugar. Natural sources of sugar, such as syrups and dried fruit, are limited to 1 tablespoon per day, and the program encourages the consumption of 1 tablespoon of ground flax seeds daily.

The program does not prescribe portion size, calorie limits, or nutrient intake goals. The participants are dissuaded from counting calories and counselled to eat to satisfy their hunger.

**Figure 2:**
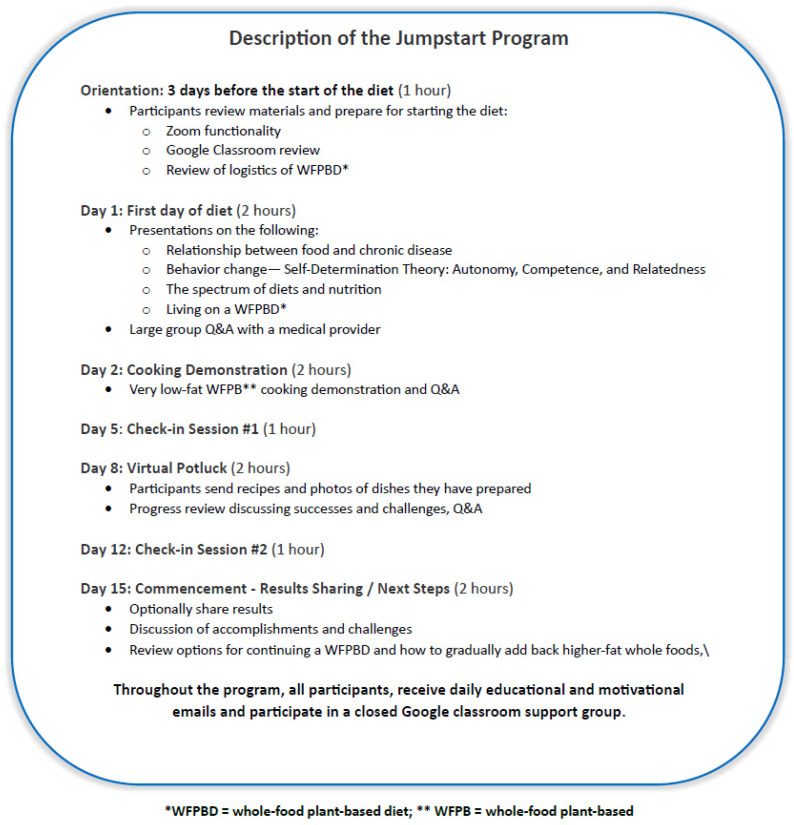
Outline of the basic structure/curriculum of the Jumpstart program.

During the program, there are discussions, cooking demonstrations, and potluck sessions where participants share successes, struggles, thoughts, accomplishments, and recipes. The participants are encouraged to ask questions during check-in sessions and in the online forum. Adherence is at the participants’ discretion, and they may self-report their adherence and deviations during the check-in sessions, but there is no formal mechanism to assess adherence to the plant-based eating program. At the end of the program, the participants are encouraged to share their feedback. Figure 2 outlines the structure and curriculum of the Jumpstart program.

### 2.3. Control Group

The control group received usual care, other than the tests required by the study protocol. At the conclusion of the study, the control subjects could attend Jumpstart at no cost, if desired.

### 2.4. Measurements

#### 2.4.1. Anthropometrics

Anthropometric measurements were performed at the start and the end of this study. Height was measured using a wall-mounted stadiometer. Weight was recorded using a medical electronic scale, Scale-Tronix, Model 6700 (Welch Allyn Inc., Skaneateles Falls, NY, USA). Body mass index (BMI) was calculated as kg/m^2^.

#### 2.4.2. Blood Pressure

Blood pressure was measured in a seated position after resting for 5–10 min using the CARESCAPE V100 monitor (GE Healthcare, Chicago, IL, USA). The subjects’ arms were measured to ensure the proper cuff size. Blood pressure medication start, withdrawal, and adjustment over the two-week period were at the discretion of the patient’s medical team and not part of the research protocol.

#### 2.4.3. Laboratory Tests

Blood testing was conducted at the start, on day eight, and at the end of the Jumpstart program for sodium, potassium, chloride, bicarbonate, blood urea nitrogen (BUN), creatinine, glucose, calcium, phosphorus, albumin, and estimated glomerular filtration rate (eGFR) and at the start and end for cholesterol, magnesium, erythrocyte sedimentation rate, fibroblast growth factor 23 (FGF-23), and parathyroid hormone. GFR was estimated using the 2021 CKD-EPI Creatinine eGFR Equation [15]. Urine was assayed for 8-isoprostane, a biomarker of oxidative stress. The testing was performed at the equivalent times for the controls.

#### 2.4.4. Other Measurements

The subjects completed a 3-day food record prior to intervention and during Jumpstart (or the equivalent times for the controls). The participants received a recording form and verbal and written instruction to record all foods and beverages consumed. Portion size was measured or estimated using visual aids. A research dietitian (N.W.) provided instruction and reviewed the completed records with the participants to improve the report quality. The data were entered into the Nutrition Data System for Research (NSDR, Nutrition Coordinating Center, University of Minnesota, Twin Cities) to calculate the nutrient intake.

The subjects received the Kidney Disease Quality-of-Life Questionnaire at the beginning and end of this study.

### 2.5. Statistical Analysis

The patient, disease, and treatment characteristics of the study population were summarized overall and by randomization arm using the counts and proportions for the categorical variables and the means and standard deviations for the continuous variables. The characteristics were compared between the treatment arms using Fisher’s exact test for the categorical variables and *t*-tests for the continuous variables. Paired *t*-tests were used to compare the changes in diet, BP, and biochemistry within the randomization groups, and two-sample *t*-tests were used to compare the within-group changes between the study arms. All the *p*-values were two-sided, with *p* ≤ 0.05 being considered statistically significant. All the analyses were conducted using SAS v 9.4 (SAS Institute, Inc., Cary NC, USA).

## 3. Results

**Figure 3:**
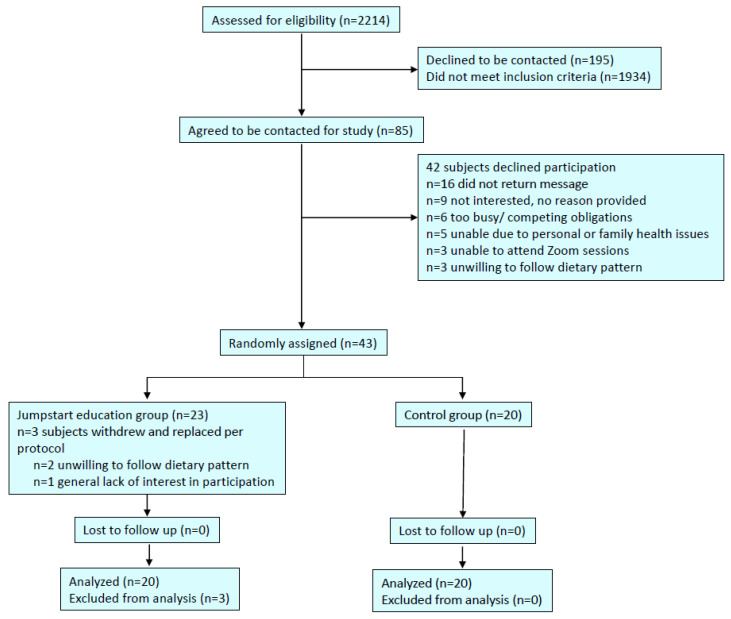
Flowchart leading into study.

The details of the patient screening, enrollment/randomization, and follow-up are in Figure 3. Three patients assigned to the intervention group withdrew and were replaced per protocol to achieve 20 participants per group. Two subjects withdrew due to concerns over the restrictiveness of the dietary interventions, and one due to lack of interest in participation. They did not attend any of the Jumpstart sessions, and each only completed the first planned assessment. These subjects were excluded from the analysis.

Table 1 shows the baseline characteristics for the groups. There were no significant differences between the groups at the baseline.

Table 2 reports the changes in the consumption of various food groups. Compared to the control group, the intervention group had a significantly higher intake of fruit, vegetables, legumes, and whole grains and a significantly decreased intake of fruit juice, refined grains, eggs, meat, dairy, and seafood.

Table 3 reports the changes in the dietary composition. The intervention group showed significant decreases in energy consumption (43%), the percentage of calories from fat (84%) and protein (27%), animal protein (>99%), cholesterol (>99%), phosphorus (22%), calcium (30%), and sodium (51%) from the baseline to the final measurement. Significant increases were seen in the intake of vegetable protein (31%), the percentage of calories from carbohydrates (64%), dietary fiber (94%), and potassium (32%). The control group had an increase in the percentage of calories from fat. The changes in dietary intake were significantly different between the intervention and control groups for all the components noted above except calcium.

Table 4 shows the primary end point results and changes in anthropometric values. At the end of the intervention, systolic blood pressure decreased by 8 mm Hg (132.7 to 124.7) in the intervention group (*p* = 0.14) and increased by 2.7 mm Hg (133.2 to 135.9) in the controls (*p* = 0.53), resulting in a 10.7 mm Hg difference in the SBP change between the intervention and control groups (*p* = 0.12). Diastolic blood pressure was not different either between or within the groups. During the study period, one subject in the intervention group required one additional anti-hypertensive medication, whereas two subjects had BP medications deprescribed (one medication for one subject and two for another). One control patient had one medication discontinued. For the remainder of the patients in the study who maintained the same blood pressure medications over the two weeks, there were no dosage adjustments.

Potassium increased by 0.01 mEq/L in the intervention group and decreased by 0.07 mEq/L in the controls. The differences within the groups were not statistically significant, nor was the change in potassium from the beginning to the end of the study different between the groups (*p* = 0.52). Three subjects in each group developed hyperkalemia (K^+^ > 5.1 mEq/L). All the episodes were mild (K^+^ ≤ 5.3 mEq/L) and resolved spontaneously.

The differences in the changes of body mass (−3.0 vs. −0.1 kg, *p* < 0.0001) and BMI (−1.1 vs. −0.2 kg/m^2^, *p* = 0.0004) between the intervention and control groups were highly significant, favoring the intervention group. There were no differences in the quality-of-life data or urinary 8-isoprostane.

Table 5 shows the changes in the biochemical values. The intervention group showed significant decreases in cholesterol, low-density lipoprotein (LDL), high-density lipoprotein (HDL), and BUN compared to the controls. FGF-23 decreased in the intervention group and increased in the controls, although this was not statistically significant (*p* = 0.07).

There were three cases of symptomatic hypotension in the intervention group. One subject noted lightheadedness at study visit 2, where their BP was 96/56 mm Hg. The subject was advised to eat more salt, and diuretics were discontinued. The symptoms resolved, and their BP improved. A second subject noted dizziness on study visit 3, where their BP was 105/68 mm Hg. Upon discussion with the treating nephrologist, diuretics were discontinued and the symptoms resolved. A third subject noted leg cramps at study visit 2, where their BP was 98/53 mm Hg. The subject was advised to drink more fluids. The cramps resolved, and their BP improved. There was one episode of acute kidney injury in the intervention group. At study visit 2, the second patient had a creatinine level of 2.66 mg/dL (baseline 2.04 mg/dL). The repeated creatinine measurement after intervention was 2.59 mg/dL. This subject showed variation in creatinine values prior to the study commencement, and 2.66 mg/dL was similar to historic values.

Several themes emerged in the feedback, which was provided by 12 of the 20 respondents. With respect to future diet plans, three out of the twelve respondents indicated they would stay on the Jumpstart diet, whereas the rest indicated some degree of incorporation of plant-based foods but did not plan on staying fully compliant. Five individuals indicated that continued support would likely be helpful in maintaining a WFPB diet. Eleven participants who provided feedback anticipated barriers, most commonly that the eating pattern promoted by the program was too restrictive, and adopting this eating pattern made eating away from home—at friends, family, or a restaurant—challenging.

## 4. Discussion

Our study demonstrates that individuals with non-diabetic, non-proteinuric CKD 3/4 who attended Jumpstart made significant dietary changes and had a non-statistically significant SBP reduction compared to the controls, without an increased risk of hyperkalemia.

Our study demonstrated a 10.7 mm Hg difference in the systolic blood pressure change between the two groups in only 14 days. Although this did not achieve statistical significance, our proof-of-concept study may have been underpowered. This degree of difference is clinically significant, and larger trials are warranted. The 8 mm Hg decrease in SBP in our intervention subjects is of a similar magnitude to that consistently seen in previous Jumpstart cohorts [13,16,17]. Our subjects’ blood pressure readings were lower than anticipated with an average systolic blood pressure in the 132–134 mm Hg range at the baseline, which may have muted the effect of the intervention, as previous non-CKD Jumpstart subjects with a baseline systolic blood pressure of >140 mm Hg saw an average reduction in systolic blood pressure of 16.8 mm Hg [13]. Nevertheless, the intervention subjects were able to reduce their blood pressure to levels nearing those suggested by practice guidelines [7]. The reduction in the intervention group also compares favorably to BP reductions seen with pharmacologic therapy [18] without the risk of blood pressure medication-related side effects.

Aside from blood pressure, our proof-of-concept study has several important findings that deserve emphasis.

The individuals undergoing the Jumpstart program made demonstrable changes in their diets, at least in the short term, which may have many positive health consequences. Our subjects clearly increased their plant protein intake, while lowering that of total and animal protein. More plant protein is a common denominator in the various healthy diet plans that, in those with CKD, have been associated with both a decrease in CKD progression and mortality [19,20]. The decrease in total protein and energy could potentially be of concern and would need to be monitored; however, several factors may reassure the clinician. While we believe that the food diary data does demonstrate significant change, underreporting on food frequency questionnaires is not uncommon [21] and may have been prevalent throughout all the subjects in our study as evidenced by the lower than expected energy and protein levels in all the queries. Reassuringly, the albumin levels were similar between our groups, and, to date, no data have shown that individuals consuming a vegan diet experience any adverse effects due to overall protein deficiency or a deficiency of any specific amino acid.

Those undergoing the education program significantly reduced their sodium intake by 51%. As noted previously, neither sodium intake nor intake of any other dietary component was specifically prescribed. It is possible that some of this decrease may be related to the decrease in overall energy, and some may be related to healthier food choices as evidenced by the increase in fruit, vegetables, whole grains, and legumes seen in the intervention group. Meta-analyses of randomized control trial data have shown that dietary sodium restriction in CKD patients leads to decreased blood pressure and decreased proteinuria/albuminuria [22,23]. Both the Kidney Disease Outcome Quality Initiative (KDOQI) and the KDIGO guidelines recommend sodium restriction (<2.3 g/day and <2 g/day, respectively [7,24]) in individuals with hypertension and CKD. As many of the top sources of sodium in the American diet are from foods that would be limited on a WFPBD [25], this approach is well suited for sodium reduction.

The intervention group also increased their dietary potassium intake by 32% without a concomitant increase in serum potassium or a difference in the number of subjects with hyperkalemia compared to the control group, despite the use of medications that may impair potassium excretion in over half of the participants. Our finding that the potassium levels and the risk of hyperkalemia did not significantly increase is concordant with the study of the DASH diet in CKD, where individuals provided with a DASH diet showed a 0.2 mEq/L increase in potassium without any new onset hyperkalemia [11]. Higher potassium intake is associated with decreased cardiovascular events and mortality in CKD patients [26,27].

Hyperkalemia is a common concern when augmenting potassium intake in CKD patients. We found that serum potassium and instances of hyperkalemia were similar between the groups, despite increased intake in the intervention group. In our study, the intervention subjects significantly increased their fruit and vegetable intake but did not significantly augment the consumption of fruit juice and decreased their consumption of meat, seafood, and dairy, all of which are high in potassium [28], illustrating that, although a WFPBD is potassium-rich, diets high in animal protein may contain significant amounts of potassium as well [29]. Food processing may further increase potassium content via potassium-containing food additives [30], such that some non-vegan diets may contain a significant amount of potassium [31]. Different methods of food preparation may also alter the potassium content. Increased potassium content may be seen after juicing, saucing, or drying fruit [29]. In addition, the relationship between dietary potassium and serum potassium in CKD is not well established [32]. One study of CKD patients showed each 10 mEq/day increase in dietary potassium led to an increase in serum potassium by 0.12 mEq/L [33].

Several factors may temper the rise in serum potassium in CKD patients who consume a whole-food plant-based diet. Alkalemia and insulin both promote the cellular uptake of potassium, and the consumption of plant-based foods, particularly fruits and vegetables, may induce insulin secretion, as well as promote a relatively more alkalemic environment [34]. While this may mitigate serum potassium increases via increased cellular uptake, ingested potassium must ultimately be excreted. In those with CKD who have a potentially reduced renal excretion of potassium, the colon can be recruited as a source of potassium excretion. Early metabolic studies have shown that, as kidney function worsens, the ability of the colon to excrete excess potassium was higher in those with CKD compared to those without [35]. Our trial suggests that augmenting potassium via a WFPBD is safe in those with moderate CKD and normokalemia, even if on medications that impair potassium excretion. Further trials are needed in more advanced CKD patients.

Dietary acid load decreased significantly in the intervention subjects. A plethora of data link high dietary acid load to the progression of chronic kidney disease and the development of ESRD [36,37,38,39], indicating that, if sustained, these changes are likely to be beneficial in our subjects with CKD and hypertension.

The total cholesterol, LDL, and HDL levels dropped significantly in the intervention subjects. While these changes appear dramatic, decreases of these magnitudes are consistent with those seen in three previous Jumpstart cohorts [13,16,17]. The finding that albumin levels were maintained in those in the intervention group argues against this being solely a marker of undernutrition. The improvements in total cholesterol and LDL levels seen in our study equal those achieved via simvastatin in the Study of Heart and Renal Protection study, which ultimately found a decrease in major atherosclerotic events in individuals with CKD [40,41]. Whereas decreases in total cholesterol and LDL can be beneficial, the impact of a decrease in HDL in plant-based diets as a predictor of cardiovascular events has been questioned [42].

The subjects in the intervention group experienced a significant decrease in body mass, while those in the control group did not. The association between obesity and hypertension has been known for decades [43]: a lower BMI and weight loss are associated with lower BP, whereas a higher BMI and weight gain show the opposite relationship [44,45,46]. Meta-analysis data implicate obesity as a risk factor for the development of hypertension [44]. Randomized controlled trial data show that WFPBDs lead to weight loss [43], as seen in our subjects. The three kilogram decrease in body mass is typical of the Jumpstart experience [13,16,17]. Obesity has also been implicated as a risk factor for CKD progression [47], although whether weight loss improves or preserves kidney function is not clear.

Phosphorus intake significantly decreased in the intervention group. Although we did not see lower serum phosphorus levels, a previous study by Moe et al. showed that plant protein led to lower phosphorus levels than animal protein for an equivalent dietary phosphorus load [48]. It is not clear whether decreased dietary phosphorus impacts long-term outcomes [24]. 

The FGF-23 levels decreased in the intervention group and increased in the control group, although the difference was not statistically significant. Our results agree with Moe et al., who found an increase in FGF-23 with a meat-heavy diet compared to a vegetarian one [48]. As FGF-23 is associated with adverse outcomes in CKD [49], this potential benefit deserves more study.

BUN decreased significantly in the intervention vs. control group, likely due to the decrease in protein consumption, but the long-term implications of this finding are uncertain. Albumin was not different between the groups.

Fiber intake was significantly increased in the intervention group but not in the control group. Increased fiber intake has been associated with improved blood pressure control in the general population [50]; decreased all-cause and cardiovascular mortality and cardiovascular disease in CKD patients [51]; and, in one small study, CKD progression [52], but interventional studies in CKD patients are lacking.

We tested an educational program rather than providing the recommended food. Goraya et al. showed that providing fruits and vegetables to individuals with CKD reduces BP [12], and our study suggests that, if food cannot be provided, an educational approach may be effective in lowering BP. As our intervention was over 15 days, further work is needed to determine the longer-term impact and sustainability of an educational approach on hypertension and outcomes such as CKD progression, cardiovascular events, and mortality.

Can dietary changes be sustained? Long-term adherence data are scarce. Discussions on adherence need to recognize the barriers to eating plant-predominant diets long-term [53], and, indeed, our patients who provided feedback noted several, namely, the restrictive nature of the dietary pattern and difficulty eating outside the home. Although limiting dietary variety may lead to decreased energy intake and weight loss [54], these benefits would likely be lost if individuals reverted back to their previous eating pattern—a risk if the dietary pattern is perceived as too restrictive. Strategies to address these concerns and to determine the support needed to sustain long-term dietary changes after programs such as Jumpstart are an area for further research.

Three patients in the intervention group experienced hypotension-related side effects. This speaks to the rapidity with which plant-based diets may decrease BP and reinforces the need for medical supervision in those planning such a change.

Our study has several limitations. The short duration cannot assess long-term outcomes. The average eGFR and BP in our cohort were higher and lower, respectively, than anticipated. Whether this approach is appropriate in those with worse metrics is unknown. Our protocol excluded people with diabetes to remove the logistics of adjusting diabetic medications over the two-week period, which may have been required due to diet-induced decreases in blood sugar [13,55]. These factors limit our study’s generalizability. Future investigations should include individuals with more severe kidney disease, higher blood pressure, and diabetes to test the efficacy and safety in these subgroups. A trial enriched with diabetic subjects would be particularly welcome as it would be important to evaluate the safety of the dietary changes with respect to glucose control, the risk of hypoglycemia, and the possible need to deprescribe medications. We also did not employ ambulatory or home BP monitoring as recommended by the KDIGO guidelines [7]. Another limitation is the potential inaccuracy of the 3-day food record, suggested by the low energy intake of both groups at the baseline and the large decrease in both energy and protein in the intervention group. We did not use an alternative way to assess adherence to a plant-based diet, and this is not tracked during the Jumpstart program. Future studies could include metabolomics, certain patterns of which have been shown to correlate to plant-based diet indices derived from FFQs and may even help distinguish a healthy plant-based dietary pattern from an unhealthy one [56], or the data from a food tracking app. Nevertheless, we believe that the marked changes in the intervention group signal true change. Finally, it is not possible to determine from our data which factors—the alteration of the dietary pattern in general or any of its specific components—were responsible for any of the positive findings over the two-week period.

## 5. Conclusions

In conclusion, we found a clinically, albeit not statistically, significant difference in SBP between the individuals with moderate CKD who participated in the Jumpstart program and those who did not, without an increased risk of hyperkalemia. The intervention patients ate more fruit, vegetables, legumes, and whole grains; significantly decreased sodium intake; increased potassium and fiber intake; and significantly decreased serum cholesterol. Studies assessing this approach over a longer duration and in individuals with more advanced and diabetic kidney disease are warranted.

## Figures and Tables

**Table 1:** Baseline characteristics of analyzed subjects.

Continuous Variables (Mean (SD))	Overall (*n* =40)	Intervention (*n* =20)	Control (*n* =20)	*p* Value
Age (mean, years)	66.9 (11.0)	68.1 (11.9)	65.7 (10.1)	0.5
Baseline GFR (mL/min)	46.0 (10.7)	45.8 (9.8)	46.2 (11.7)	0.91
Weight (kg)	87.9 (17.8)	89.8 (16.9)	86 (18.9)	0.51
Body mass index (kg/m^2^)	31.1 (5.4)	32.4 (5.9)	29.8 (4.7)	0.13
Number of blood pressure medications	2.2 (1.1)	1.9 (0.9)	2.5 (1.1)	0.07
Categorical Variables (n (%))				
Gender				0.53
Female	21 (52.5%)	12 (60.0%)	9 (45.0%)	
Male	19 (47.5%)	8 (40.0%)	11 (55.0%)	
Race				1
White	29 (72.5%)	14 (70.0%)	15 (75.0%)	
Nonwhite	11 (27.5%)	6 (30.0%)	5 (25.0%)	
Ethnicity (% Hispanic)				-
Hispanic	0	0	0	
Non-Hispanic	40 (100%)	20 (100%)	20 (100%)	
CKD cause (%)				0.19
Hypertension	25 (62.5%)	10 (50.0%)	15 (75.0%)	
Other	15 (37.5%)	10 (50.0%)	5 (25.0%)	
Coronary artery disease	6 (15.0%)	3 (15.0%)	3 (15.0%)	1
Cerebrovascular disease	2 (5.0%)	0	2 (10.0%)	0.49
Angiotensin converting enzyme inhibitor	6 (15.0%)	3 (15.0%)	3 (15.0%)	1
Angiotensin receptor blocker	11 (27.5%)	5 (25.0%)	6 (30.0%)	1
Potassium sparing diuretic	6 (15.0%)	4 (20.0%)	2 (10.0%)	0.66
Beta blocker	23 (57.5%)	14 (70.0%)	9 (45.0%)	0.2
Diuretic	18 (45.0%)	9 (45.0%)	9 (45.0%)	1

**Table 2:** Consumption of various food groups before and after this study (serving per day).

	Intervention Group		Control Group		Final vs. Baseline Difference (Mean (SD))		
	(*N* = 20, Mean (SD))		(*N* =20, Mean (SD))				
Food Group	Baseline	Final	Baseline	Final	Change in Intervention	Change in Control	*p*-Value
Fruit	2.0 (2.5)	3.4 (2.5) **	1.4 (1.5)	1.1 (1.5)	1.4 (3.4)	−0.5 (2.0)	<0.0001
Vegetables (excluding fried)	2.7 (2.6)	7.2 (4.3) ***	2.5 (2.4)	2.1 (1.9)	4.4 (4.4)	−0.4 (2.2)	<0.0001
Legumes	0.1 (0.4)	1.2 (1.3) ***	0.1 (0.2)	0.1 (0.3)	1.1 (1.3)	0.0 (0.4)	<0.0001
Nuts and seeds	23.1 (5.6)	15.5 (4.2)	0.8 (2.0)	1.0 (2.9)	−0.2 (1.1)	0.2 (3.5)	0.07
Fruit juice	0.1 (0.6) †	0.0 (0.1)	0.5 (1.0)	0.5 (0.9)	−0.1 (0.6)	−0.0 (1.1)	0.0005
Whole grains	1.2 (1.5)	2.7 (2.1) ***	1.6 (2.1)	1.2 (1.6)	1.5 (2.6)	−0.4 (1.7)	<0.0001
Refined grains	4.4 (3.1)	0.7 (1.1) ***	3.6 (3.3)	3.6 (2.5)	−3.7 (3.0)	−0.0 (3.8)	<0.0001
Eggs	0.5 (0.8)	0.0 (0.0) ***	0.4 (0.7)	0.5 (0.9)	−0.5 (0.8)	0.2 (0.9)	<0.0001
Meat (including poultry)	2.9 (3.1)	0.0 (0.0) ***	2.5 (3.5)	2.5 (2.5)	−2.9 (3.1)	−0.1 (3.3)	<0.0001
Dairy	2.7 (3.2)	0.0 (0.1) ***	3.1 (3.2)	2.4 (2.1)	−2.6 (3.2)	−0.7 (3.0)	<0.0001
All seafood	0.7 (2.3)	0.0 (0.0) *	0.7 (1.5)	1.1 (2.3)	−0.7 (2.3)	0.4 (2.6)	0.0004

* *p* < 0.05 between baseline and final; ** *p* < 0.01 between baseline and final; and *** *p* < 0.0001 between baseline and final. † *p* < 0.05 between intervention baseline and control baseline

**Table 3:** Food diary data for intervention and control groups (reported as daily intake).

	Intervention Group (*n* =20, Mean (SD))		Control Group (*n* =20, Mean (SD))		Final vs. Baseline Difference (Mean (SD))		
Variable	Baseline	Final	Baseline	Final	Change in Intervention	Change in Control	*p*-Value
Energy (kcal)	1828.1 (439.9)	1051.1 (254.3) **	1677.8 (654.1)	1708.8 (431.4)	−776.9 (370.0)	31.0 (399.8)	<0.0001
Energy/kg	20.9 (5.7)	12.5 (3.7) **	19.8 (7.8)	20.4 (6.2)	−8.4 (4.5)	0.7 (4.9)	<0.0001
Total fat (g)	73.5 (27.1)	11.9 (6.3) **	66.7 (37.3)	73.8 (22.1)	−61.7 (25.7)	7.1 (27.5)	<0.0001
% calories from fat	34.9 (8.2)	9.6 (4.0) **	33.1 (7.5)	37.4 (5.8) *	−25.3 (9.3)	4.3 (7.2)	<0.0001
% calories from carbohydrates	47.8 (10.0)	78.2 (5.5) **	49.4 (11.1)	46.6 (6.5)	30.3 (10.2)	−2.8 (8.9)	<0.0001
% calories from protein	15.1 (2.7)	11.1 ** (1.8)	15.2 (4.5)	15.3 (3.3)	−4.0 (3.5)	0.1 (3.9)	0.001
Total protein (g)	68.9 (25.4)	35.6 (14.0) **	63.7 (32.5)	65.4 (26.6)	33.3 (27.8)	1.6 (33.3)	<0.0001
Total protein (g/kg)	0.8 (0.4)	0.4 (0.2) **	0.8 (0.4)	0.8 (0.4)	−0.4 (0.4)	0.0 (0.0)	<0.0001
Animal protein (g)	41.9 (16.8)	0.2 (0.6) **	36.7 (25.4)	38.9 (17.0)	−41.7 (16.9)	2.2 (20.7)	<0.0001
Vegetable protein (g)	27.0 (8.4)	35.4 (9.4) *	27.1 (13.9)	26.5 (15.6)	8.4 (8.6)	−0.6 (11.9)	0.01
Cholesterol (mg)	239.1 (90.5)	0.9 (2.7) **	221.3 (151.0)	246.0 (121.5)	−238.1 (90.6)	24.8 (84.4)	<0.0001
Total dietary fiber (g)	20.3 (6.2)	39.4 (11.4) **	18.8 (9.7)	17.6 (9.3)	19.1 (12.9)	−1.2 (5.7)	<0.0001
Total fiber per 1000 Kcal	12.1 (5.3)	38.0 (8.7) **	11.7 (5.5)	10.7 (4.9)	25.8 (8.7)	−1.0 (3.2)	<0.0001
Calcium (mg)	744.2 (278.0)	523.3 (213.3) *	722.6 (401.1)	780.0 (430.2)	−220.9 (376.7)	57.3 (329.1)	0.21
Phosphorus (mg)	1058.3 (237.4)	823.4 (218.2) *	1030.1 (424.4)	1085.3 (330.3)	−234.9 (250.7)	55.2 (276.3)	0.001
Magnesium (mg)	266.6 (61.5)	364.9 (96.1) **	271.7 (121.1)	284.7 (106.5)	98.3 (90.8)	13.0 (93.4)	0.01
Sodium (mg)	2457.7 (583.5)	1193.4 (812.6) **	2377.1 (956.7)	2287.6 (654.4)	−1264.30 (998.5)	−89.5 (740.8)	0.0001
Potassium (mg)	2397.0 (465.4)	3174.6 (922.3) *	2263.7 (838.3)	2187.9 (698.1)	777.7 (929.3)	−75.8 (651.9)	0.002
Potential renal acid load (mEq)	8.1 (19.4)	−32.2 (18.3) **	7.4 (17.0)	10.6 (15.5)	−40.4 (26.1)	3.3 (21.0)	<0.001

* *p* ≤ 0.001 between baseline and final; and ** *p* ≤ 0.0001 between baseline and final.

**Table 4:** Baseline and final blood pressure values, serum potassium levels, and body mass data of participants.

	Intervention Group		Control Group		Final vs. Baseline Difference (Mean (SD))		
	(*n* =20, Mean (SD))		(*n* =20, Mean (SD))				
Variable	Baseline	Final	Baseline	Final	Change in Intervention	Change in Control	*p*-Value
SBP (mm Hg)	132.7 (21.4)	124.7 (13.9)	133.2 (16.0)	135.9 (15.7)	−8.0 (23.3)	2.7 (19.0)	0.12
DBP (mm Hg)	68.7 (10.7)	69.6 (9.5)	73.1 (8.4)	73.3 (7.9)	1.0 (12.0)	0.2 (7.6)	0.81
Potassium (mEq/L)	4.4 (0.5)	4.4 (0.4)	4.3 (0.5)	4.3 (0.4)	0.0 (0.5)	−0.1 (0.4)	0.52
Body mass (kg)	89.8 (16.9)	86.8 (16.9)	86.0 (18.9)	85.9 (18.7)	−3.0 (1.1)	−0.1 (1.0)	<0.0001
Body mass index (kg/m^2^)	32.4 (5.9)	31.3 (5.9)	29.8 (4.7)	29.6 (4.6)	−1.1 (0.4)	−0.2 (0.9)	0.0004

Abbreviations: SBP = systolic blood pressures; and DBP = diastolic blood pressure.

**Table 5:** Other relevant biochemical data.

	Intervention Group		Control Group		Final vs. Baseline Difference (Mean (SD))		
	(*N* =20, Mean (SD))		(*N* =20, Mean (SD))				
Variable	Baseline	Final	Baseline	Final	Change in Intervention	Change in Control	*p*-Value
Sodium (mEq/L)	139.5 (2.4)	138.0 (3.2)	140.2 (1.9)	140.2 (2.4)	−1.5 (3.2)	0 (1.9)	0.08
Potassium (mEq/L)	4.4 (0.5)	4.4 (0.4)	4.3 (0.5)	4.3 (0.4)	0.0 (0.5)	−0.1 (0.4)	0.52
Chloride (mEq/L)	102.1 (2.4)	100.3 (3.8)	103.0 (2.4)	102.8 (2.6)	−1.9 (3.7)	−0.2 (1.9)	0.08
Bicarbonate (mEq/L)	25.2 (4.5)	25.4 (3.3)	25.7 (2.0)	25.1 (2.6)	0.15 (4.1)	−0.7 (2.2)	0.45
BUN (mg/dL)	23.1 (5.6)	15.5 (4.2)	23.1 (6.8)	22.4 (6.6)	−7.6 (5.3)	−0.8 (4.4)	<0.0001
Creatinine (mg/dL)	1.5 (0.3)	1.6 (0.4)	1.6 (0.6)	1.6 (0.6)	0.10 (0.22)	0.03 (0.14)	0.24
Glucose (mg/dL)	98.3 (11.0)	98.3 (8.9)	99.2 (11.7)	96.6 (11.3)	−0.05 (11.4)	−2.55 (9.3)	0.45
Calcium (mg/dL)	9.7 (0.42)	9.7 (0.4)	9.6 (0.4)	9.6 (0.5)	−0.03 (0.3)	0.01 (0.3)	0.71
Phosphorus (mg/dL)	3.6 (0.43)	3.6 (0.6)	3.4 (0.6)	3.6 (0.7)	−0.03 (0.5)	0.2 (0.7)	0.26
PTH (pg/mL)	67.3 (29.2)	74.1 (40.3)	66.8 (25.6)	66.7 (31.0)	6.9 (18.5)	−0.1 (20.7)	0.27
Albumin (g/dL)	4.4 (0.3)	4.5 (0.3)	4.4 (0.3)	4.4 (0.3)	0.04 (0.2)	−0.04 (0.2)	0.28
Magnesium (mEq/L)	2.1 (0.2)	2.2 (0.3)	2.1 (0.2)	2.1 (0.3)	0.05 (0.2)	0.01 (0.2)	0.50
ESR (mm/h)	21.1 (13.4) (*N* =19)	18.8 (13.5)	18.0 (20.5)	18.7 (21.1)	−2.5 (9.4)	0.7 (5.3)	0.21
FGF-23 (RU/mL)	61.4 (33.6) (*N* =19)	56.5 (24.1)	65.1 (40.9)	68.6 (46.0) (*N* =18)	−5.6 (22.2)	8.4 (23.3)	0.07
Total cholesterol (mg/dL)	195.3 (63.2)	158.1 (50.1)	175.5 (42.7)	170.6 (39.0)	−37.3 (27.5)	−5.0 (18.7)	0.0001
LDL (mg/dL)	107.3 (57.7)	81.2 (47.1)	94.4 (34.9)	93.8 (31.1)	−26.1 (22.7)	−0.6 (12.4)	0.0001
HDL (mg/dL)	58.3 (20.3)	49.7 (14.5)	53.4 (16.1)	53.0 (17.0)	−8.6 (8.1)	−0.4 (5.1)	0.0006
Triglycerides (mg/dL)	136.6 (66.9)	136.9 (68.6)	131.1 (82.1)	122.2 (53.4)	0.25 (80.0)	−9.0 (49.7)	0.67
Urinary 8-isoprostane (ng/mg of creat)/kg	0.02 (0.02) (*N* =19)	0.02 (0.02) (*N* =19)	0.02 (0.02)	0.02 (0.01)	0.003 (0.009) (*N* =19)	−0.003 (0.02)	0.24

Abbreviations: BUN = blood urea nitrogen; PTH = parathyroid hormone; ESR = erythrocyte sedimentation rate; FGF = fibroblast growth factor; LDL = low-density lipoprotein; and HDL = high-density lipoprotein.

## Data Availability

The data are available on request by emailing the authors.

## References

[1] United States Renal Data System (2022). 2022 USRDS Annual Data Report: Epidemiology of Kidney Disease in the United States.

[2] Lv J., Ehteshami P., Sarnak M.J., Tighiouart H., Jun M., Ninomiya T., Foote C., Rodgers A., Zhang H., Wang H. (2013). Effects of intensive blood pressure lowering on the progression of chronic kidney disease: A systematic review and meta-analysis. CMAJ.

[3] Cheung A.K., Rahman M., Reboussin D.M., Craven T.E., Greene T., Kimmel P.L., Cushman W.C., Hawfield A.T., Johnson K.C., Lewis C.E. (2017). Effects of Intensive BP Control in CKD. J. Am. Soc. Nephrol..

[4] Wright J.T., Williamson J.D., Whelton P.K., Snyder J.K., Sink K.M., Rocco M.V., Reboussin D.M., Rahman M., Oparil S., Lewis C.E. (2015). A Randomized Trial of Intensive versus Standard Blood-Pressure Control. N. Engl. J. Med..

[5] Aggarwal R., Petrie B., Bala W., Chiu N. (2019). Mortality Outcomes With Intensive Blood Pressure Targets in Chronic Kidney Disease Patients. Hypertension.

[6] Malhotra R., Nguyen H.A., Benavente O., Mete M., Howard B.V., Mant J., Odden M.C., Peralta C.A., Cheung A.K., Nadkarni G.N. (2017). Association Between More Intensive vs Less Intensive Blood Pressure Lowering and Risk of Mortality in Chronic Kidney Disease Stages 3 to 5: A Systematic Review and Meta-analysis. JAMA Intern. Med..

[7] (2021). KDIGO 2021 Clinical Practice Guideline for the Management of Blood Pressure in Chronic Kidney Disease. Kidney Int..

[8] Whelton P.K., Carey R.M., Aronow W.S., Casey D.E., Collins K.J., Himmelfarb C.D., DePalma S.M., Gidding S., Jamerson K.A., Jones D.W. (2018). 2017 ACC/AHA/AAPA/ABC/ACPM/AGS/APhA/ASH/ASPC/NMA/PCNA Guideline for the Prevention, Detection, Evaluation, and Management of High Blood Pressure in Adults: A Report of the American College of Cardiology/American Heart Association Task Force on Clinical Practice Guidelines. Hypertension.

[9] Appel L.J., Moore T.J., Obarzanek E., Vollmer W.M., Svetkey L.P., Sacks F.M., Bray G.A., Vogt T.M., Cutler J.A., Windhauser M.M. (1997). A Clinical Trial of the Effects of Dietary Patterns on Blood Pressure. N. Engl. J. Med..

[10] Joshi S., Ettinger L., Liebman S.E. (2019). Plant-Based Diets and Hypertension. Am. J. Lifestyle Med..

[11] Tyson C.C., Lin P.-H., Corsino L., Batch B.C., Allen J., Sapp S., Barnhart H., Nwankwo C., Burroughs J., Svetkey L.P. (2016). Short-term effects of the DASH diet in adults with moderate chronic kidney disease: A pilot feeding study. Clin. Kidney J..

[12] Goraya N., Simoni J., Jo C.-H., Wesson D.E. (2013). A Comparison of Treating Metabolic Acidosis in CKD Stage 4 Hypertensive Kidney Disease with Fruits and Vegetables or Sodium Bicarbonate. Clin. J. Am. Soc. Nephrol..

[13] Friedman S.M., Barnett C.H., Franki R., Pollock B., Garver B., Barnett T.D. (2022). Jumpstarting Health With a 15-Day Whole-Food Plant-Based Program. Am. J. Lifestyle Med..

[14] Esselstyn C.B. (1999). Updating a 12-year experience with arrest and reversal therapy for coronary heart disease (an overdue requiem for palliative cardiology). Am. J. Cardiol..

[15] Inker L.A., Eneanya N.D., Coresh J., Tighiouart H., Wang D., Sang Y., Crews D.C., Doria A., Estrella M.M., Froissart M. (2021). New Creatinine- and Cystatin C-Based Equations to Estimate GFR without Race. N. Engl. J. Med..

[16] Friedman S.M., Scheuer K., Beha B.G., Dewhirst M., Barnett T.D. (2023). Whole-food plant-based Jumpstart for a Deaf and Hard of Hearing cohort. Front. Nutr..

[17] Nyong F.A., Barnett T.D., Garver B., Dewhirst M., Pollock B., Friedman S.M. (2023). A whole-food, plant-based program in an African American faith-based population. Front. Nutr..

[18] Zanchetti A., Thomopoulos C., Parati G. (2015). Randomized Controlled Trials of Blood Pressure Lowering in Hypertension. Circ. Res..

[19] Hu E.A., Coresh J., Anderson C.A.M., Appel L.J., Grams M.E., Crews D.C., Mills K.T., He J., Scialla J., Rahman M. (2020). Adherence to Healthy Dietary Patterns and Risk of CKD Progression and All-Cause Mortality: Findings From the CRIC (Chronic Renal Insufficiency Cohort) Study. Am. J. Kidney Dis..

[20] Kelly J.T., Palmer S.C., Wai S.N., Ruospo M., Carrero J.-J., Campbell K.L., Strippoli G.F.M. (2017). Healthy Dietary Patterns and Risk of Mortality and ESRD in CKD: A Meta-Analysis of Cohort Studies. Clin. J. Am. Soc. Nephrol..

[21] Bedard D., Shatenstein B., Nadon S. (2004). Underreporting of energy intake from a self-administered food-frequency questionnaire completed by adults in Montreal. Public Health Nutr..

[22] Garofalo C., Borrelli S., Provenzano M., De Stefano T., Vita C., Chiodini P., Minutolo R., De Nicola L., Conte G. (2018). Dietary Salt Restriction in Chronic Kidney Disease: A Meta-Analysis of Randomized Clinical Trials. Nutrients.

[23] McMahon E.J., Campbell K.L., Bauer J.D., Mudge D.W., Kelly J.T. (2021). Altered dietary salt intake for people with chronic kidney disease. Cochrane Database Syst. Rev..

[24] Ikizler T.A., Burrowes J.D., Byham-Gray L.D., Campbell K.L., Carrero J.J., Chan W., Fouque D., Friedman A.N., Ghaddar S., Goldstein-Fuchs D.J. (2020). KDOQI Clinical Practice Guideline for Nutrition in CKD: 2020 Update. Am. J. Kidney Dis..

[25] Ahmed M., Ng A.P., Christoforou A., Mulligan C., L’Abbé M.R. (2023). Top Sodium Food Sources in the American Diet-Using National Health and Nutrition Examination Survey. Nutrients.

[26] Leonberg-Yoo A.K., Tighiouart H., Levey A.S., Beck G.J., Sarnak M.J. (2017). Urine Potassium Excretion, Kidney Failure, and Mortality in CKD. Am. J. Kidney Dis..

[27] Suh S.H., Song S.H., Oh T.R., Choi H.S., Kim C.S., Bae E.H., Oh K.H., Lee J., Han S.H., Kim Y.H. (2021). Association of Urinary Potassium Excretion with Blood Pressure Variability and Cardiovascular Outcomes in Patients with Pre-Dialysis Chronic Kidney Disease. Nutrients.

[28] U.S. Department of Agriculture, U.S. Department of Health and Human Services (2020). Dietary Guidelines for Americans, 2020–2025.

[29] Palmer B.F., Colbert G., Clegg D.J. (2020). Potassium Homeostasis, Chronic Kidney Disease, and the Plant-Enriched Diets. Kidney360.

[30] Parpia A.S., L’Abbé M., Goldstein M., Arcand J., Magnuson B., Darling P.B. (2018). The Impact of Additives on the Phosphorus, Potassium, and Sodium Content of Commonly Consumed Meat, Poultry, and Fish Products Among Patients With Chronic Kidney Disease. J. Ren. Nutr..

[31] Allès B., Baudry J., Méjean C., Touvier M., Péneau S., Hercberg S., Kesse-Guyot E. (2017). Comparison of Sociodemographic and Nutritional Characteristics between Self-Reported Vegetarians, Vegans, and Meat-Eaters from the NutriNet-Santé Study. Nutrients.

[32] Babich J.S., Kalantar-Zadeh K., Joshi S. (2022). Taking the Kale out of Hyperkalemia: Plant Foods and Serum Potassium in Patients With Kidney Disease. J. Ren. Nutr..

[33] Ogata S., Akashi Y., Kato S., Oka Y., Suda A., Yoshizaki S., Maeda Y., Nishimura K., Maeda K., Nakai S. (2023). Association Between Dietary Potassium Intake Estimated From Multiple 24-Hour Urine Collections and Serum Potassium in Patients With CKD. Kidney Int. Rep..

[34] St-Jules D.E., Goldfarb D.S., Sevick M.A. (2016). Nutrient Non-equivalence: Does Restricting High-Potassium Plant Foods Help to Prevent Hyperkalemia in Hemodialysis Patients?. J. Ren. Nutr..

[35] Hayes C., McLeod M., Robinson R. (1967). An extravenal mechanism for the maintenance of potassium balance in severe chronic renal failure. Trans. Assoc. Am. Physicians.

[36] Banerjee T., Crews D.C., Wesson D.E., Tilea A.M., Saran R., Ríos-Burrows N., Williams D.E., Powe N.R. (2015). High Dietary Acid Load Predicts ESRD among Adults with CKD. J. Am. Soc. Nephrol..

[37] Crews D.C., Banerjee T., Wesson D.E., Morgenstern H., Saran R., Burrows N.R., Williams D.E., Powe N.R. (2018). Race/Ethnicity, Dietary Acid Load, and Risk of End-Stage Renal Disease among US Adults with Chronic Kidney Disease. Am. J. Nephrol..

[38] Kanda E., Ai M., Kuriyama R., Yoshida M., Shiigai T. (2014). Dietary acid intake and kidney disease progression in the elderly. Am. J. Nephrol..

[39] Toba K., Hosojima M., Kabasawa H., Kuwahara S., Murayama T., Yamamoto-Kabasawa K., Kaseda R., Wada E., Watanabe R., Tanabe N. (2019). Higher estimated net endogenous acid production with lower intake of fruits and vegetables based on a dietary survey is associated with the progression of chronic kidney disease. BMC Nephrol..

[40] Sharp Collaborative G. (2010). Study of Heart and Renal Protection (SHARP): Randomized trial to assess the effects of lowering low-density lipoprotein cholesterol among 9438 patients with chronic kidney disease. Am. Heart J..

[41] Wang F., Zheng J., Yang B., Jiang J., Fu Y., Li D. (2015). Effects of Vegetarian Diets on Blood Lipids: A Systematic Review and Meta-Analysis of Randomized Controlled Trials. J. Am. Heart Assoc..

[42] Kent L., Morton D., Hurlow T., Rankin P., Hanna A., Diehl H. (2013). Long-term effectiveness of the community-based Complete Health Improvement Program (CHIP) lifestyle intervention: A cohort study. BMJ Open.

[43] Kannel W.B., Brand N., Skinner J.J., Dawber T.R., McNamara P.M. (1967). The relation of adiposity to blood pressure and development of hypertension. The Framingham study. Ann. Intern. Med..

[44] Jayedi A., Rashidy-Pour A., Khorshidi M., Shab-Bidar S. (2018). Body mass index, abdominal adiposity, weight gain and risk of developing hypertension: A systematic review and dose-response meta-analysis of more than 2.3 million participants. Obes. Rev..

[45] Stevens V.J., Obarzanek E., Cook N.R., Lee I.M., Appel L.J., Smith West D., Milas N.C., Mattfeldt-Beman M., Belden L., Bragg C. (2001). Long-term weight loss and changes in blood pressure: Results of the Trials of Hypertension Prevention, phase II. Ann. Intern. Med..

[46] Yang S., Zhou Z., Miao H., Zhang Y. (2023). Effect of weight loss on blood pressure changes in overweight patients: A systematic review and meta-analysis. J. Clin. Hypertens..

[47] Chang A.R., Grams M.E., Ballew S.H., Bilo H., Correa A., Evans M., Gutierrez O.M., Hosseinpanah F., Iseki K., Kenealy T. (2019). Adiposity and risk of decline in glomerular filtration rate: Meta-analysis of individual participant data in a global consortium. BMJ.

[48] Moe S.M., Zidehsarai M.P., Chambers M.A., Jackman L.A., Radcliffe J.S., Trevino L.L., Donahue S.E., Asplin J.R. (2011). Vegetarian Compared with Meat Dietary Protein Source and Phosphorus Homeostasis in Chronic Kidney Disease. Clin. J. Am. Soc. Nephrol..

[49] Fliser D., Kollerits B., Neyer U., Ankerst D.P., Lhotta K., Lingenhel A., Ritz E., Kronenberg F., Group f.t.M.S. (2007). Fibroblast Growth Factor 23 (FGF23) Predicts Progression of Chronic Kidney Disease: The Mild to Moderate Kidney Disease (MMKD) Study. J. Am. Soc. Nephrol..

[50] Reynolds A.N., Akerman A., Kumar S., Diep Pham H.T., Coffey S., Mann J. (2022). Dietary fibre in hypertension and cardiovascular disease management: Systematic review and meta-analyses. BMC Med..

[51] Gai W., Lin L., Wang Y., Bian J., Tao Y. (2024). Relationship between dietary fiber and all-cause mortality, cardiovascular mortality, and cardiovascular disease in patients with chronic kidney disease: A systematic review and meta-analysis. J. Nephrol..

[52] Lu L., Huang Y.F., Wang M.Q., Chen D.X., Wan H., Wei L.B., Xiao W. (2017). Dietary fiber intake is associated with chronic kidney disease (CKD) progression and cardiovascular risk, but not protein nutritional status, in adults with CKD. Asia Pac. J. Clin. Nutr..

[53] Fehér A., Gazdecki M., Véha M., Szakály M., Szakály Z. (2020). A Comprehensive Review of the Benefits of and the Barriers to the Switch to a Plant-Based Diet. Sustainability.

[54] Raynor H.A. (2012). Can limiting dietary variety assist with reducing energy intake and weight loss?. Physiol. Behav..

[55] Barnard N.D., Cohen J., Jenkins D.J.A., Turner-McGrievy G., Gloede L., Jaster B., Seidl K., Green A.A., Talpers S. (2006). A Low-Fat Vegan Diet Improves Glycemic Control and Cardiovascular Risk Factors in a Randomized Clinical Trial in Individuals with Type 2 Diabetes. Diabetes Care.

[56] Kim H., Yu B., Li X., Wong K.E., Boerwinkle E., Seidelmann S.B., Levey A.S., Rhee E.P., Coresh J., Rebholz C.M. (2022). Serum metabolomic signatures of plant-based diets and incident chronic kidney disease. Am. J. Clin. Nutr..

